# Democratising Early Career Researcher (ECR) Training for Dementia Research

**DOI:** 10.1002/alz.093305

**Published:** 2025-01-09

**Authors:** Sarah Bauermeister

**Affiliations:** ^1^ University of Oxford, Oxford, Oxfordshire United Kingdom

## Abstract

**Background:**

Training programmes and workshops for Early Career Researchers (ECR) in dementia research are frequently expensive and inaccessible. This means that under resourced institutions, researchers and countries (LMIC) are unable to provide adequate training facilities for researchers focused on analyses for dementia outcomes. The result is that only elite institutions are provided high level training facilities for their researchers and those who are not represented in these institutions are underrepresented. Dementias Platform UK (DPUK) provides free and affordable training opportunities for all academic and industry researchers across the world.

**Method:**

The DPUK training programme includes:

1. Datathon workshops which enable researchers from across the world to access large cohort datasets on the DPUK Data Portal and work alongside other global researchers on a dementia‐focused research question.

2. Academies for both elementary and advanced longitudinal data analysis are conducted twice a year.

3. A 12‐week mentoring programme is provided after the Datathon workshop ‐ led by DPUK and aimed at producing a publishable output from their Datathon attendance.

**Result:**

Over 200 researchers across 11 countries have attended the DPUK Training events over the last 5 years.

**Conclusion:**

Providing affordable or free training for the current and next generation of ECRs is possible by using the resources provided by a Trusted Research Environment (TRE) such as DPUK.

